# Intraoperative protective mechanical ventilation: what is new?

**DOI:** 10.5935/0103-507X.20170065

**Published:** 2017

**Authors:** Luiz Marcelo Sá Malbouisson, Raphael Augusto Gomes de Oliveira

**Affiliations:** 1 Surgical Intensive Care Units, Hospital das Clínicas, Faculdade de Medicina, Universidade de São Paulo - São Paulo (SP), Brazil.; 2 Discipline of Anesthesiology, Hospital das Clínicas, Faculdade de Medicina, Universidade de São Paulo - São Paulo (SP), Brazil.; 3 Intensive Care Unit, Hospital Sírio-Libanês - São Paulo (SP), Brazil.

## Introduction

Postoperative pulmonary complications are an important cause of hospital morbidity and mortality and are known to be associated with longer hospital stays and higher long-term mortality rates.^(1)^ Thus, it is imperative to recognize early risk factors for the development of postoperative pulmonary complications (PPC) and to focus on the adoption of measures to prevent them from occurring.^(1)^ Among these measures, recent evidence points to some generally defined strategies, such as intraoperative protective mechanical ventilation, that may help minimize the occurrence of PPC. Other methods include the rational use of the fraction of inspired oxygen (FiO_2_), tidal volume (Vt) and positive end-expiratory pressure (PEEP).^(2)^

## Risk factors for postoperative pulmonary complications

Currently, a number of risk factors related to the development of PPCs are known; they may be associated with the patient, surgical procedure and/or anesthetic management. Based on recent evidence, the Assess Respiratory Risk in Surgical Patients in Catalonia (ARISCAT)^(3)^ is believed to be the best tool for the preoperative identification of patients at risk of developing PPCs^(2,4)^ (Table 1).

**Table 1:** Assess Respiratory Risk in Surgical Patients in Catalonia predictive scores

Variables	Scoring
Age (years)	
≤ 50	0
51 - 80	3
> 80	16
Preoperative SpO_2_ (%)	
≥ 96	0
91 - 95	8
≤ 90	24
Respiratory infection in the last month	
No	0
Yes	17
Preoperative anemia (Hemoglobin ≤ 10g/dL)	
No	0
Yes	11
Surgical incision	
Peripheral	0
Abdominal	15
Intrathoracic	24
Duration of surgery (hours)	
< 2	0
2 - 3	16
> 3	23
Emergency surgery	
No	0
Yes	8

SpO_2_ - pulse oximetry; Low risk < 26 points: predicted rate of postoperative pulmonary complications of 0.87%; intermediate risk 26 - 44 points: predicted rate of postoperative pulmonary complications of 7.82%; high risk ≥ 45 points: predicted rate of postoperative pulmonary complications of 38.1%.^(4)^

## Fraction of inspired oxygen

In humans, the indiscriminate use of high FiO_2_ may lead to direct pulmonary toxicity and the development of interstitial fibrosis, reabsorption atelectasis and tracheobronchitis.^(5)^ In addition, hyperoxia is associated with increased production of reactive oxygen species, which cause damage to cellular structures in animal models.^(5)^ In a randomized clinical trial in patients undergoing abdominal surgery, the use of high FiO_2_ (80%) in the perioperative period was not associated with an increase in the rates of pulmonary complications and hospital mortality compared to a low FiO_2_ group (30%), although mortality at 30 days was statistically higher in the subgroup of patients who underwent colorectal surgery using a high FiO_2_ strategy.^(6)^

Recently, data from a randomized clinical trial that assessed the role of hyperoxia in the outcomes of critically ill patients brought further controversy to the deleterious effects of hyperoxia, although the study was terminated early due to recruitment difficulties. In a group of critically ill patients treated with a hyperoxic strategy (arterial partial pressure of oxygen - PaO_2_ > 150mmHg), there were higher mortality rates in the intensive care unit, including cases of circulatory shock, hepatic dysfunction and bacteremia, compared to a group treated with the conservative strategy (PaO_2_ 70 - 100mmHg).^(5)^

Thus, the lowest possible FiO_2_ is usually recommended to prevent hypoxia and to avoid hyperoxia. Although there is no robust evidence for recommendations in all groups of surgical patients, using the lowest possible FiO_2_ to maintain a peripheral arterial saturation (SpO_2_) level above 92% is recommended in non-obese surgical patients with healthy lungs undergoing open abdominal surgery.^(7)^

## Tidal volume

Historically, high Vt values (up to 15mL/kg predicted body weight - PBW) were used during the anesthetic act in order to increase the end-expiratory lung volume and to reduce the incidence of atelectasis,^(8)^ although such relationships were not effectively demonstrated in a clinical trial using computed tomography of the chest.^(9)^ However, as was already robustly demonstrated in critically ill patients,^(10)^ the use of low Vt values is associated with a reduction in lung injuries induced by mechanical ventilation and has been consistently described as more appropriate for pulmonary protection during the intraoperative period.^(11)^ This rationale is based on three large randomized clinical trials that demonstrated that intraoperative ventilation with a Vt of 6 - 8mL/kg PBW prevents the development of PPC in patients undergoing elective surgery.^(12-14)^

In addition, there is currently an association between higher distending pressure values (defined by the difference between the plateau pressure and the PEEP), which correspond to the Vt values corrected for complacency of the respiratory system, and worse clinical outcomes in patients with acute respiratory distress syndrome.^(15)^ Although there are no randomized clinical trials evaluating this strategy in the context of intraoperative surgical patients, a recent meta-analysis of individual data has shown that intraoperative ventilation in patients undergoing elective surgeries with high distending pressure values, as well as changes in PEEP values that promote an increase in distending pressure, is associated with the development of PPCs.^(15)^

Thus, it is believed that patients with healthy lungs subjected to intraoperative ventilation during open abdominal surgery benefit from a Vt of 6 to 8mL/kg PBW.^(12-14)^ Further evidence is still needed to recommend intraoperative ventilation based on distending pressure. However, it is worth noting that the potential deleterious effect of high distending pressures in this scenario should be avoided, suggesting that the plateau and PEEP pressures should be routinely monitored during the intraoperative period.^(16)^

## Positive end-expiratory pressure and alveolar recruitment maneuvers

The use of PEEP during intraoperative mechanical ventilation is based on the idea of maintaining open alveoli during the respiratory cycle and on the opening of atelectatic areas due to mechanical ventilation and the anesthetic act.^(2)^ On the other hand, the strategy of intraoperative permissive atelectasis, in which PEEP levels are kept low without alveolar recruitment maneuvers, aims to minimize stress on the pulmonary epithelium.^(2)^

Currently, there is evidence that the use of PEEP can reduce atelectasis, improve compliance without increasing dead space, and maintain the end expiratory volume in obese and non-obese patients under general anesthesia.^(2)^ However, a recently published randomized controlled trial compared mechanical ventilation with a Vt of 8mL/kg PBW and a low PEEP strategy (≤ 2cmH_2_O) without alveolar recruitment maneuvers to a high PEEP strategy (PEEP 12cmH_2_O) with alveolar recruitment maneuvers in non-obese patients undergoing elective open abdominal surgery. There were no notable differences in PPCs between the two groups. However, the high PEEP group had higher rates of intraoperative arterial hypotension and a greater need for vasoactive drugs compared to the low PEEP group.^(7)^

Thus, it is believed that patients with healthy lungs undergoing mechanical ventilation during open abdominal surgery benefit from PEEP values of up to 2cmH_2_O without the use of alveolar recruitment maneuvers. In cases of hypoxemia with no response to increased FiO_2_ and PEEP, alveolar recruitment maneuvers based on the gradual increase in the Vt may be used.^(7)^

## Conclusion

**Figure 1:**
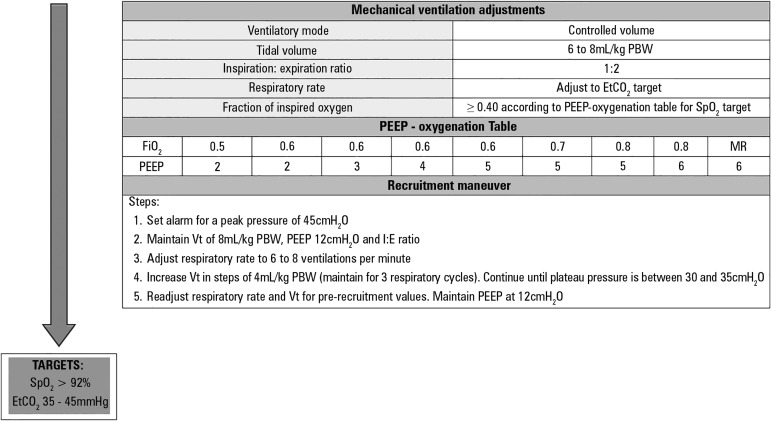
Suggested algorithm for mechanical ventilation in patients with healthy lungs undergoing open abdominal surgery. PBW - predicted body weight, calculated based on the predefined formula: 50 + 0.91 x (height in cm - 152.4) for men and 45.5 + 0.91 x (height in cm - 152.4) for women; EtCO_2_ - carbon dioxide partial pressure at end of expiration; PEEP - positive end-expiratory pressure; SpO_2_ - peripheral arterial saturation; FiO_2_ - fraction of inspired oxygen; RM - recruitment maneuver; Vt - tidal volume; I:E - inspiration:expiration ratio.

Adapted from: PROVE Network Investigators for the Clinical Trial Network of the European Society of Anaesthesiology, Hemmes SN, Gama de Abreu M, Pelosi P, Schultz MJ. High versus low positive end-expiratory pressure during general anaesthesia for open abdominal surgery (PROVHILO trial): a multicentre randomised controlled trial. Lancet 2014;384(9942):495-503.^(7)^

The adoption of protective intraoperative ventilatory strategies is critical to the reduction of postoperative pulmonary complications. Currently, based on the best scientific evidence available, the use of low Vt values, which is associated with low PEEP and FiO_2_ values, appears to be the best strategy available for minimizing complications and improving clinical outcomes (Figure 1).
